# Building a better bacillus: the emergence of *Mycobacterium tuberculosis*

**DOI:** 10.3389/fmicb.2014.00139

**Published:** 2014-04-03

**Authors:** Joyce Wang, Marcel A. Behr

**Affiliations:** ^1^Department of Microbiology and Immunology, McGill UniversityMontreal, QC, Canada; ^2^Department of Medicine, McGill UniversityMontreal, QC, Canada; ^3^McGill International TB CentreMontreal, QC, Canada

**Keywords:** *Mycobacterium tuberculosis*, *Mycobacterium kansasii*, *M. tuberculosis*-specific genes, horizontal gene transfer, comparative genomics

## Abstract

The genus *Mycobacterium* is comprised of more than 150 species that reside in a wide variety of habitats. Most mycobacteria are environmental organisms that are either not associated with disease or are opportunistic pathogens that cause non-transmissible disease in immunocompromised individuals. In contrast, a small number of species, such as the tubercle bacillus, *Mycobacterium tuberculosis*, are host-adapted pathogens for which there is no known environmental reservoir. In recent years, gene disruption studies using the host-adapted pathogen have uncovered a number of “virulence factors,” yet genomic data indicate that many of these elements are present in non-pathogenic mycobacteria. This suggests that much of the genetic make-up that enables virulence in the host-adapted pathogen is already present in environmental members of the genus. In addition to these generic factors, we hypothesize that molecules elaborated exclusively by professional pathogens may be particularly implicated in the ability of *M. tuberculosis* to infect, persist, and cause transmissible pathology in its host species, *Homo sapiens*. One approach to identify these molecules is to employ comparative analysis of mycobacterial genomes, to define evolutionary events such as horizontal gene transfer (HGT) that contributed *M. tuberculosis*-specific genetic elements. Independent studies have now revealed the presence of HGT genes in the *M. tuberculosis* genome and their role in the pathogenesis of disease is the subject of ongoing investigations. Here we review these studies, focusing on the hypothesized role played by HGT loci in the emergence of *M. tuberculosis* from a related environmental species into a highly specialized human-adapted pathogen.

## INTRODUCTION

Through modification of their genome content, bacteria can evolve to exploit different ecological niches. While vertical events such as gene duplication, chromosomal rearrangement and gene decay can affect the shape and structure of a genome (Ventura et al., 2007), horizontal gene transfer (HGT) is an important mechanism for bacteria to acquire novel genetic material into their genomes (Lercher and Pal, 2008; Price et al., 2008), subsequently facilitating adaptation and diversification (Treangen and Rocha, 2011). HGT can be mediated by transformation (acquisition of naked DNA), transduction (DNA transfer via a bacteriophage), and conjugation (fusion of two bacterial cells enabling a unidirectional transfer of a plasmid or mobile element; Frost et al., 2005). HGT has been shown to profoundly impact the prokaryotic genome plasticity, allowing the acquisition of antibiotic resistance elements (Mohd-Zain et al., 2004; Palmer et al., 2010; Gray et al., 2013), virulence genes (Rosas-Magallanes et al., 2006; Li et al., 2012) and new metabolic pathways (Wilson et al., 2003; Baldwin et al., 2004; Chouikha et al., 2006; Noda-Garcia et al., 2013).

*Mycobacterium*, a genus of Actinobacteria, comprises mostly non-pathogenic species. Exceptionally, this genus contains a number of host-adapted pathogens, including the leprosy bacillus, *Mycobacterium leprae*, and the Johne’s bacillus, *M. avium* subspecies* paratuberculosis*, the latter defined by the presence of at least six genomic islands that were likely acquired by HGT (Alexander et al., 2009). In this review, we focus on the *Mycobacterium tuberculosis* complex (MTBC), agents of tuberculosis (TB) in their respective mammalian hosts. Among the various subspecies of the MTBC, *M. tuberculosis sensu stricto* is the cause of human TB, which infects over 2 billion people and causes an estimated 1.3 million deaths annually (World Health Organization, 2013).

When contrasting the genome of MTBC organisms with the most closely related environmental mycobacteria, *M. marinum* and *M. kansasii*, independent studies have identified *M. tuberculosis-*specific genetic factors putatively acquired by HGT (Becq et al., 2007; Veyrier et al., 2009; Supply et al., 2013), evidenced by the presence of clustering, vehicles of HGT (phage, transposons, toxin-antitoxin genes) and an aberrant GC content in their DNA (Zaneveld et al., 2008). For example, the *M. tuberculosis* genome codes for 55 proteins absent from *M. kansasii, M. marinum* and all other sequenced mycobacterial genomes (Veyrier et al., 2009). As 87% of these *M. tuberculosis*-specific genes are found in clusters, it has been postulated that these clusters may be pathogenicity islands that contribute to the unique virulence of* M. tuberculosis* (Hacker et al., 1997; Veyrier et al., 2009). As several of these *M. tuberculosis*-specific genes have been linked to host adaptation (Sassetti and Rubin, 2003; Pethe et al., 2004), this provides further support for the notion that HGT may have played a crucial role in the emergence of this pathogen. At the ecological level, *M. tuberculosis* uses humans as its sole known reservoir while environmental mycobacteria such as *M. kansasii* can be found in various aquatic habitats (McSwiggan and Collins, 1974; Steadham, 1980; Sartori et al., 2013; Thomson et al., 2013), further highlighting the impact of genome remodeling on bacterial biology.

**FIGURE 1:**
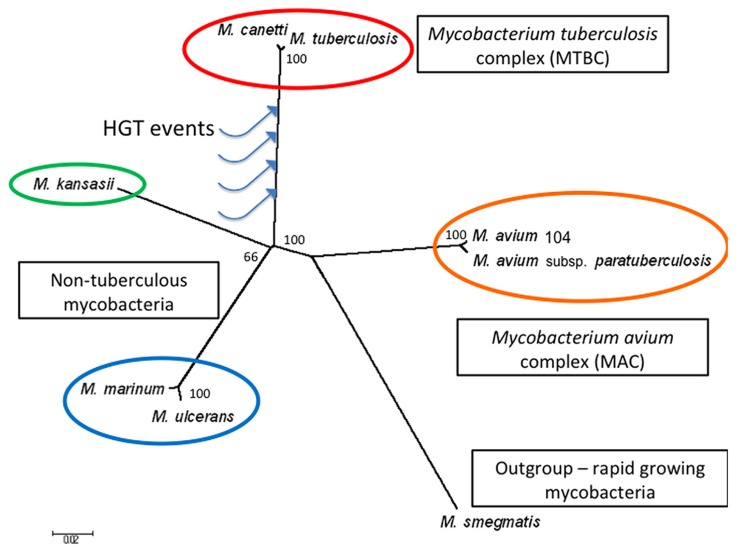
**Phylogeny of *M. tuberculosis* and closely related *Mycobacterium* species**. The un-rooted phylogenetic tree was generated by MEGA6.0 using 20 randomly selected genes conserved across eight *Mycobacterium* species (Schwab et al., 2009). The blue arrows schematically represent where putative HGT events may have occurred, resulting in *M. tuberculosis*-specific genomic islands. The scale bar indicates 0.02 substitutions per nucleotide position, and the bootstrap values calculated using the neighbor-joining method (expressed as a percentage of 1000 replicates) are shown at the branch points. The fast growing species *M. smegmatis* is used as the out-group. Genes used are listed below (represented as *M. tuberculosis* genes): *Rv0001*-*dnaA, Rv0041*-*leuS, Rv0236A*–*Rv0236A, Rv0248c*–*Rv0248c, Rv0285*-*PE5, Rv0287*-*esxG, Rv0288*-*esxH, Rv1085c*–*Rv1085c, Rv0197*–*Rv0197, Rv1304*-*atpB, Rv1305*-*atpE, Rv1894c*–*Rv1894c, Rv2172c*–*Rv2172c, Rv2392*-*cysH, Rv2440c*-*obg, Rv2477c*–*Rv2477c, Rv3019c*-*esxR, Rv3045*-*adhC, Rv3392c*-*cmaA1, Rv3502c*- *hsd4A*.

**FIGURE 2:**
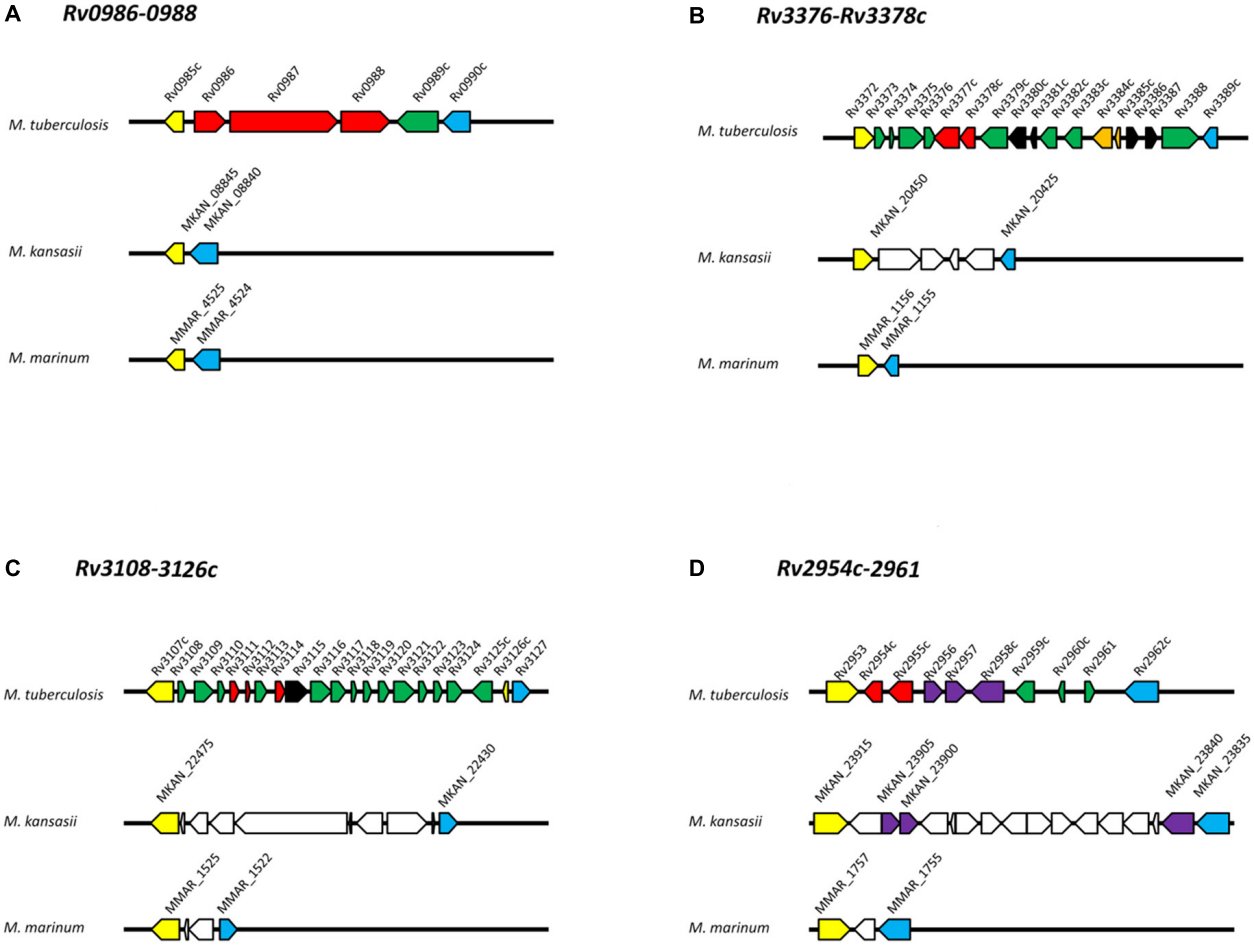
**Genomic organization of operons in *M. tuberculosis, M. kansasii*, and *M. marinum.* (A)** *Rv0986-0988*; **(B)** *Rv3376-3378c*; **(C)** *Rv3108-3126c*; **(D)** *Rv2954c-2961*. Protein-coding genes are represented by arrows and orthologous genes are indicated by arrows of the same color. Yellow and blue arrows mark the “boundary” of each *M. tuberculosis-*specific locus. Red arrows indicate genes discussed in this review. Dark green arrows indicate *M. tuberculosis* genes with no orthologs within the corresponding *M. kansasii* and *M. marinum* genomic regions. White arrows in *M. kansasii* and *M. marinum* genomes indicate genes present but not orthologs to *M. tuberculosis* genes. Black arrows indicate transposases; orange arrows indicate toxin-antitoxin genes. Genome organizations for *M. tuberculosis, M. kansasii, M. marinum* and gene clusters were obtained from the Kyoto Encyclopedia of Genes and Genomes (http://www.kegg.jp/) based on databases available at the Sanger Institute, Tuberculist, and McGill University, respectively. Orthologs were verified by comparing each predicted protein against the H37Rv genome using the BLAST program. Only proteins with >50% coverage, 60% identity and E-value <e^-20^ were used.

In this review, we briefly describe the early interplay between *M. tuberculosis* and the host during an infection, followed by bioinformatic data supporting the evidence for HGT and its potential contribution to the host-adapted lifestyle of this pathogen. To illustrate the relationships between various mycobacteria including the species discussed in this manuscript, in **Figure 1** we present an un-rooted phylogenic tree based on 20 randomly selected genes conserved across eight mycobacteria, including seven slow-growing species (*M. tuberculosis, M. canetti, M. kansasii, M. marinum, M. ulcerans, M. avium* subsp. *hominssuis, M. avium* subsp. *paratuberculosis*) and a rapid-growing species (*M. smegmatis*) as the out-group. It is worth noting that the topology of this independently generated tree is congruent with the tree built from housekeeping genes (Veyrier et al., 2009), providing additional support for the evolutionary relationships between these species. The genes used for tree generation are provided in the figure legend. The organization of each *M. tuberculosis*-specific locus discussed is illustrated in **Figure 2**, in comparison with the flanking genomic regions in the related organisms *M. kansasii* and *M. marinum*.

## *M. tuberculosis* AND THE HOST ENVIRONMENT

When *M. tuberculosis* enters the pulmonary alveoli via the aerosol route, it is thought to first encounter alveolar macrophages. Following phagocytosis by these macrophages, the bacterium finds itself in the phagosomal compartment which is, among other attributes, iron limiting, carbon poor, hypoxic, nitrosative, and oxidative (Schnappinger et al., 2003). *M. tuberculosis* is able to resist these bactericidal effects by synthesizing antioxidants, repairing DNA and proteins, maintaining intracellular pH and cell wall integrity (Buchmeier et al., 2000; Master et al., 2002; Boshoff et al., 2003; Darwin and Nathan, 2005; Vandal et al., 2008, 2009; Colangeli et al., 2009). Moreover, *M. tuberculosis* can cope with hypoxic and other growth-limiting environments using a number of tactics such as activating the dormancy regulon, promoting alternate metabolic pathways and iron metabolism (Leistikow et al., 2010; Marrero et al., 2010; Ryndak et al., 2010; Griffin et al., 2011). In addition, *M. tuberculosis* is able to prevent the fusion of phago-lysosome (Sun et al., 2010; Wong et al., 2011), permeabilize the phagosomal membrane (Manzanillo et al., 2012), and escape into the cytosol. The cytosol likely offers a less hostile, thus more permissive environment, where the bacteria can replicate and induce the infected macrophages to undergo necrosis instead of apoptosis, a strategy that allows the bacteria to infect neighboring cells, thereby enabling the perpetuation of the infection process (van der Wel et al., 2007; Divangahi et al., 2009; Behar et al., 2010).

### *Rv*0986–0988

In the seminal work that first described evidence of HGT in the *M. tuberculsosis* genome, Becq et al. (2007) detected a 5.6 kb *M. tuberculosis*-specific Island with a reduced GC content (53%) compared to the average for the *M. tuberculosis* genome (65.6%; Cole et al., 1998). Further molecular and *in silico* analyses demonstrated that this operon is present in other members of the MTBC, including *M. bovis, M. africanum,* and *M. microti* (Rosas-Magallanes et al., 2006). Based on phylogenetic analyses, it was proposed that three genes within this locus, *Rv0986–8*, had been acquired from phylogenetically distant γ-proteobacteria via plasmid transfer. The fact that the orthologs of these three genes are consistently together suggest that one single HGT event occurred during the acquisition of this operon by the ancestor of *M. tuberculosis* (Rosas-Magallanes et al., 2006).

*Rv0986* is predicted to encode an adenosine triphosphate (ATP)-binding protein that is orthologous to the *Agrobacterium tumefaciens attE* polypeptide, and form an ABC transporter with *Rv0987* (Braibant et al., 2000; Rosas-Magallanes et al., 2006). The *A. tumefaciens attE* gene is located on a plasmid which harbors the *attE-H* operon. This operon has been proposed to encode an ABC transporter that secretes a host cell adhesion factor (Matthysse et al., 1996, 2000). Intriguingly, the N- and C- terminal sequences of *Rv0987* share 40% similarity with *attF* and *attG*, and the neighboring *Rv0988* shows 50% similarity with *attH* (Rosas-Magallanes et al., 2006).

The *Rv0986–8* operon has been implicated in *M. tuberculosis* virulence as mutants with disruption in *Rv0986* and *0987* exhibit reduced ability to inhibit phagosome acidification in macrophages (Pethe et al., 2004). Furthermore, these mutants had impaired binding to host cells, and this phenotype could be rescued by complementing with a cosmid carrying *M. tuberculosis* DNA encompassing the *Rv0986–8* operon (Rosas-Magallanes et al., 2007). Although *Rv0986* and *Rv0987* mutants were not shown to be attenuated in mouse lungs and spleens (Rosas-Magallanes et al., 2007), the *Rv0986* mutant was subsequently shown to be less virulent in the context of central nervous system infection (Be et al., 2008). Recently *Rv0986–8* have been found to be regulated by EspR, a transcription factor that also regulates the ESX-1 secretion system (Blasco et al., 2012), a major virulence mediator of *M. tuberculosis* (Brodin et al., 2006).

### *Rv*3376–*Rv*3378c

Another genomic island potentially acquired by HGT is the 3.1 kb region encompassing *Rv3376–8*c (Becq et al., 2007; Veyrier et al., 2009). This island exhibits a reduced GC content (54.7%) and is associated with the presence of transposases, known to mediate HGT events (Becq et al., 2007; Veyrier et al., 2009). The closest genera harboring such genes are *Agrobacterium* and *Rhizobium* (Becq et al., 2007). More recently, Mann and Peters, (2012) speculated that, while *Rv3377c* amino acid sequence shares homology with proteins from another actinomycete, *Micromonospora, Rv3378c* has no ortholog in any other organism with the exception of a hypothetical protein in amoeba. These observations suggest that these MTBC-specific genes originated in different sources (Mann and Peters, 2012).

Biochemical characterization has revelated that *Rv3377c* and *Rv3378c* encode a halimadienyl diphosphate (HPP) synthase and a diterpene synthase, respectively. In a step-wise fashion, these enzymes catalyze the cyclization of the precursor, geranylgeranyl diphosphate (GGPP), and the hydrolysis of the HPP intermediate to produce isotuberculosinol (isoTB), a diterpene species (Nakano et al., 2005; Mann et al., 2009a, b). Terpenes are one of the most widespread and chemically diverse compounds found in nature. They are hydrocarbons made up of five, or multiples of five, carbon units (Simion, 2005). In plants and fungi, they are commonly found as essential as well as secondary metabolites involved in signaling and defense (Buckingham, 1994). While members of the Actinobcteria group synthesize a plethora of natural products (Baltz, 2008), very few are known to encode diterpene (C_20_) synthases (Dairi et al., 2001; Hamano et al., 2002; Dürr et al., 2006; Smanski et al., 2011).

During macrophage infection, *M. tuberculosis* mutants with disruption in either *Rv3377c* or *Rv3378c* showed marked defect in arresting phagosome acidification as well as intracellular survival, suggesting that these genes are involved in the modulation of early infection process (Pethe et al., 2004). Intriguingly, these genes are not transcriptionally altered during macrophage infection (Stewart et al., 2005; Rohde et al., 2007; Waddell and Butcher, 2007), implying that the synthesis of isoTB is regulated at the protein level, potentially triggered by ambient magnesium levels (Mann et al., 2009a, 2011). In addition, isoTB has shown to inhibit phagosome acidification by 0.5 pH units as well as proteolytic activity (Mann et al., 2009b). Recently *Rv3378c* has been characterized as a tuberculosinyl transferase that converts isoTB into the proposed end product, tuberculosinyladenosine (TbAd; Layre et al., 2014). The cellular mechanism by which *Rv3377–8c* modifies phagosome function thus remains to be further investigated.

### *Rv*3108–3126c

*Rv3108–3126c* is a 15.1 kb, MTBC-specific genomic island that has a reduced GC content (56.7%) and contains genes encoding insertion sequences and a transposase, features typical of HGT (Becq et al., 2007). The proposed donor species include *Burkholderia, Corynebacterium*, and *Pseudomonas*. This island contains two potential virulence genes; *Rv3111* and *Rv3114*.

A transposon mutant of *Rv3111* (*moaC1*) was first shown to be attenuated in replicating in macrophages (Rosas-Magallanes et al., 2007). In a more recent high-throughput genetic screen, mutants disrupted for the genes *moaC1*/*moaD1*(*Rv3112*), implicated in molybdenum cofactor biosynthesis, were found to be trafficked to acidified intracellular compartments rapidly (Brodin et al., 2010), potentially providing an explanation for the impaired intracellular growth. Molybdopterin is the main building block of the molybdenum cofactor (MoCo) and can be found in enzymes that catalyze redox reactions in carbon, nitrogen, and sulfur utilization (Williams et al., 2011). *moeB1*, a homologous gene potentially involved in MoCo biosynthesis, has also been shown to be required for arresting phagosome acidification (MacGurn and Cox, 2007). *moaC1* mutant itself has exhibited reduced virulence in macrophages as well as primate lungs (Rosas-Magallanes et al., 2007; Dutta et al., 2010). Further investigation of how MoCo-mediated redox reactions alter the intraphagosomal environment should provide more insight on the cellular processes employed by *M. tuberculosis* to adapt to the mammalian host environment.

*Rv3114* has been shown to be required for *M. tuberculosis* persistence in the mouse spleen (Sassetti and Rubin, 2003) and is temporally regulated during infection (Talaat et al., 2004). It encodes a putative nucleoside deaminase involved in nucleotide metabolism (Akhter et al., 2008).

### *Rv*2954c–2961

*Rv2954c–2961* make-up a 7 kb genomic island with a low GC content (53.6%) and a transposase gene (Becq et al., 2007). A phylogenetic analysis of multiple mycobacterial genome sequences proposed a step-wise acquisition of the genes within this locus. Specifically, some genes are present in slow-growing, but not rapid-growing mycobacteria, suggesting that they were acquired by the common ancestor of the slow-growing species. Conversely, other genes in this island are specific to *M. tuberculosis* and are therefore inferred to have been acquired after the common ancestor with *M. kansasii* and* M. marinum* (Veyrier et al., 2009).

Genes within this island are involved in the synthesis and modification of phenolic glycolipids (PGLs), complex lipids located in the outermost layer of the mycobacterial cell envelope. PGLs are composed of long-chain fatty acid backbones with a phenol ring and methylated sugars, including two rhamnosyl and a terminal fucosyl residue (Onwueme et al., 2005). PGLs are only produced by the members of the MTBC and related slow-growing mycobacteria, yet even among the mycobacteria that make PGL; there are species-specific modifications in the carbohydrate moiety [Daffe and Laneelle, 1988; Onwueme et al., 2005; schematically illustrated in (Veyrier et al., 2011)]. The PGLs have been implicated in mycobacterial pathogenicity such as oxidative stress resistance (Chan et al., 1989), cell tropism (Ng et al., 2000; Rambukkana, 2001), and immunomodulation (Reed et al., 2004; Guenin-Mace et al., 2009; Cambier et al., 2014).

The major type of PGLs produced by *M. tuberculosis* is denoted PGL-tb (Simeone et al., 2007). While most genes involved in the synthesis of the lipid core and carbohydrates are characterized, the enzymes responsible for *O*-methylating the fucosyl residue remained elusive until recently. It is now known that *Rv2954c*–*Rv2956* code for the methyltransferases that are responsible for *O*-methylation of the terminal fucosyl residue (Veyrier et al., 2009; Simeone et al., 2013). These three proteins catalyze the *O*-methylation of the hydroxyl groups of the terminal fucosyl residue of PGL-tb in a sequential process (Simeone et al., 2013).

In other pathogenic mycobacteria that lack the *Rv2954c, Rv2955c*, and *Rv2956* orthologs such as *M. marinum* and *M. leprae*, their PGLs do not contain the terminal O-methylated fucosyl residue (Onwueme et al., 2005). Although *M. kansasii* does not possess *Rv2954c* or *2955c*, it does encode an enzyme that is highly similar to *Rv2956* (84%), and its PGL contains four sugar residues (Riviere et al., 1987; Onwueme et al., 2005). Interestingly, *Rv2954c* and *Rv2955c* have been found to be virulence genes during macrophage infection (Rosas-Magallanes et al., 2007), and *Rv2954c* was induced upon exposure to lung surfactant (Schwab et al., 2009).

As the enzymes encoded within this island have been observed to catalyze the transfer of functional groups from one molecule to another, they may play an important role at “decorating” existing mycobacterial products and fine-tuning host responses toward the organism to optimize its intracellular survival (Veyrier et al., 2011).

## CONCLUSION

Phylogenetic analyses have been used as robust and reliable tools for identifying potential HGT loci in *M. tuberculosis* and other pathogenic mycobacteria. However, the biological relevance of most of these genomic regions remains to be delineated. In this review we examine four examples of how such putative HGT genes can affect the physiology of the pathogen and its interaction with the host. The functional characterization of these and other putative HGT-associated genes will allow us to understand whether and how HGT events have contributed to the pathogenesis of *M. tuberculosis*, ultimately guiding the development of new diagnostic tests and vaccines against this particularly successful pathogen.

## Conflict of Interest Statement

The authors declare that the research was conducted in the absence of any commercial or financial relationships that could be construed as a potential conflict of interest.
